# Supplementary figures and images for: The ability of transcription factors to differentially regulate gene expression is a crucial component of the mechanism underlying inversion, a frequently observed genetic interaction pattern
Source: PLoS Comput Biol. 2019 May 13;15(5):e1007061. doi: 10.1371/journal.pcbi.1007061 (PMC6532943; doi:10.1371/journal.pcbi.1007061)

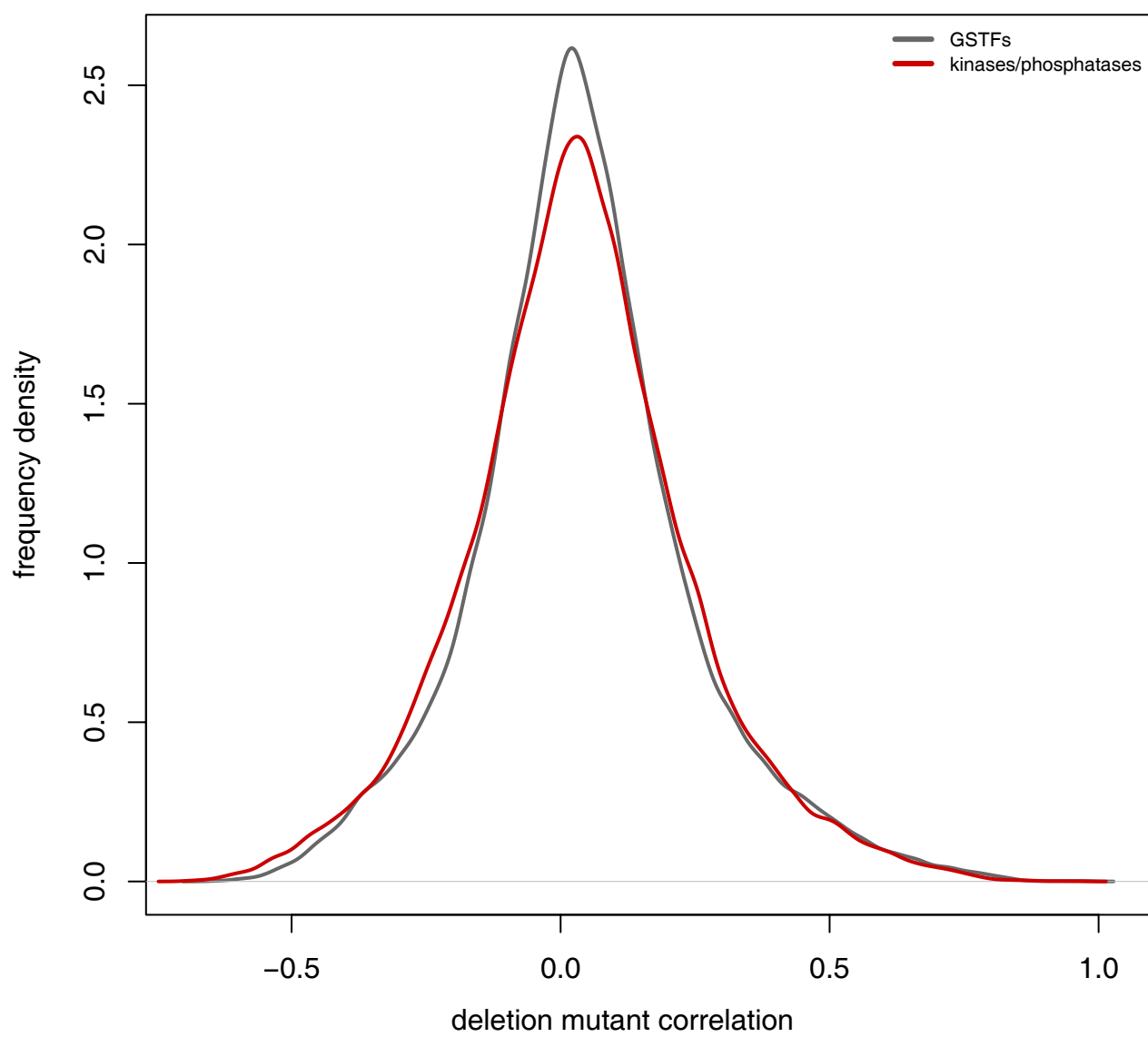

Supplement: S1 Fig — Frequency density distribution of correlations between expression profiles of pairs involving at least one GSTF (black) or kinase/phosphatase (red). Expression profiles and corresponding correlations between pairs are obtained from [47]. (PDF) [file pcbi.1007061.s003.pdf]

A

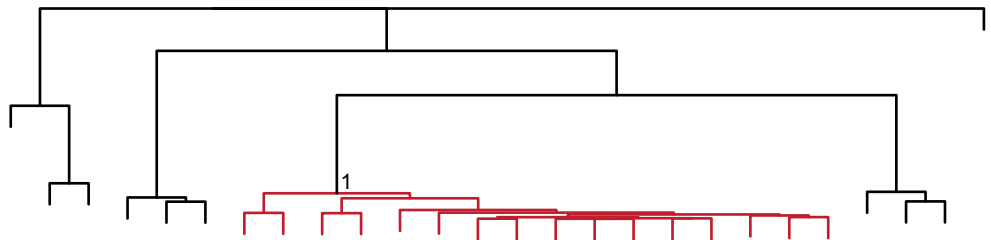

B

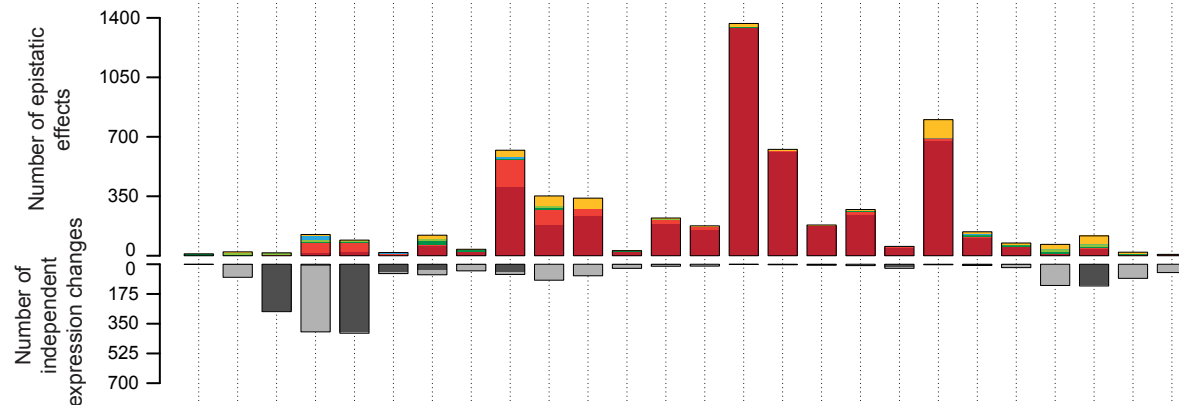

Supplement: S2 Fig — (A) Hierarchical clustering of all pairs according to their genetic interaction effects. Average linkage clustering was applied to group pairs with similar genetic interaction patterns. The number of occurrences for each genetic interaction pattern was used and the identity of individual genes was disregarded. Similarity between pairs was calculated using the cosine correlation. Most pairs are grouped together in a single branch (indicated in red), which is dominated by buffering. (B) The number of genetic interaction effects underlying the clustering are shown as bar plots below the dendrogram (top; colors as in Fig 1A). (B) Number of genes showing no genetic interaction pattern but significantly changing in one of the mutants compared to WT (bottom; adjusted p-value ≤ 0.01, FC > 1.5). Dark gray for the first named gene, light gray for the second named gene. (PDF) [file pcbi.1007061.s004.pdf]

**A**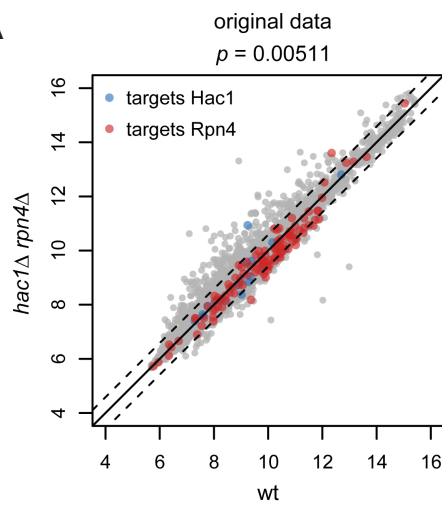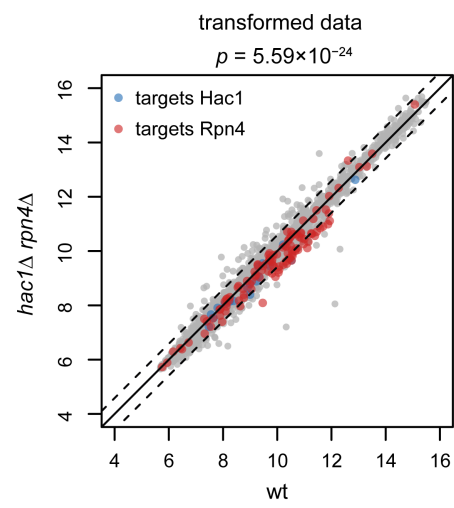**B**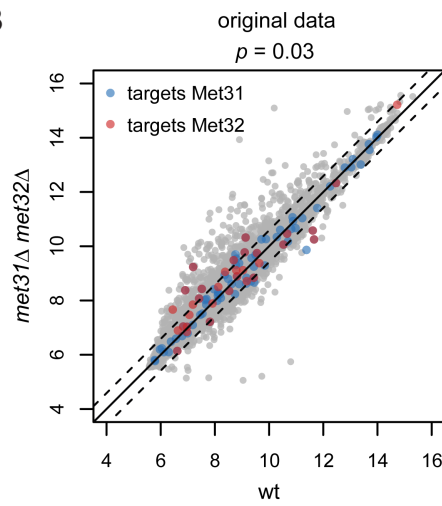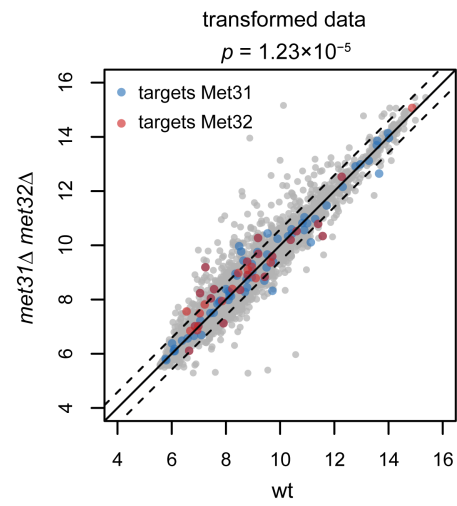**C**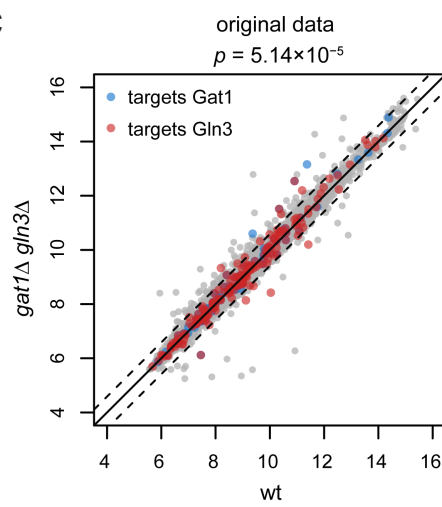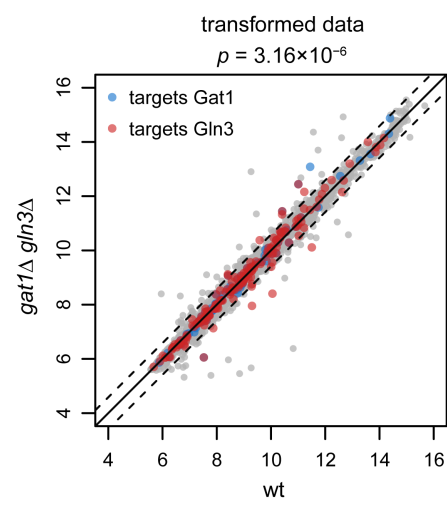**D**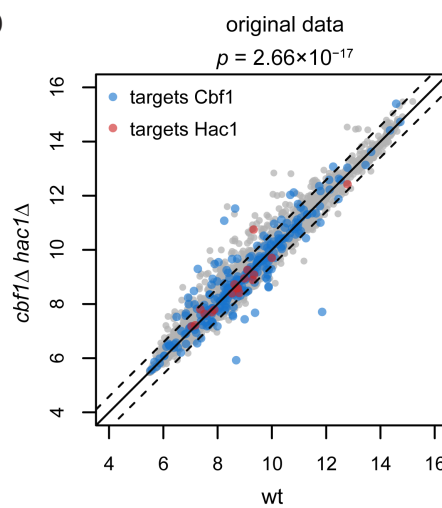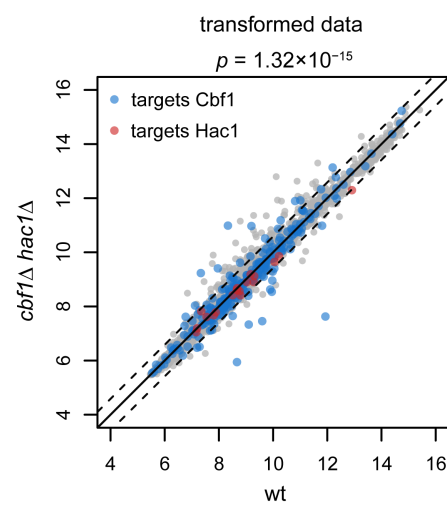

Supplement: S3 Fig — Scatter plots showing gene expression levels in the GSTF double mutant pairs hac1Δ rpn4Δ (A), met31Δ met32Δ (B), gat1Δ gln3Δ (C) and cbf1Δ hac1Δ (D) versus WT before (left) or after (right) slow growth correction. Individual transcripts are represented as dots. The dashed line indicates a FC of 1.7. Dots depicted in blue and red correspond to targets of the first and second gene in a named GSTF pair. Adjusted p-values are calculated using a hypergeometric testing procedure to test the enrichment of GSTF targets among genes that change more than 1.7 fold before (left) or after (right) slow growth correction. (PDF) [file pcbi.1007061.s005.pdf]

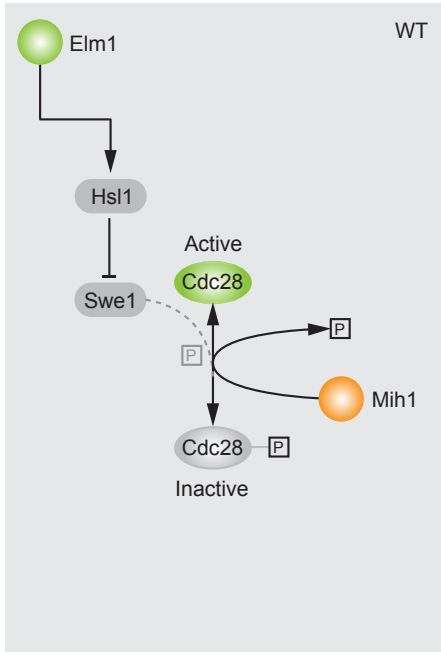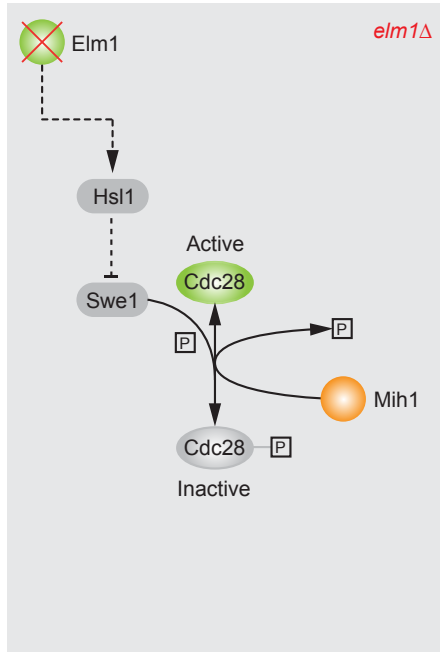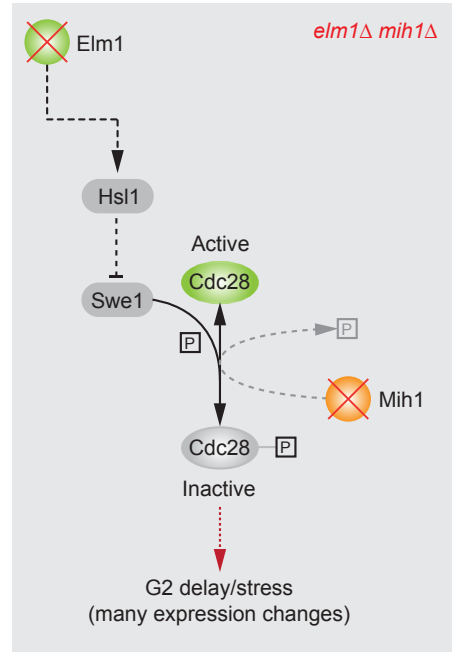

Supplement: S4 Fig — Cartoon depicting the proposed genetic interaction between Elm1 and Mih1. (left panel) WT situation where the activity of Cdc28 is not disrupted by Swe1 phosphorylation. (Middle panel) Deletion of Elm1 leads to derepression of Swe1 activity. The increase of Swe1 activity can be compensated by Mih1. (Right panel) Deletion of both Elm1 and Mih1 will cause an increase of phosphorylated Cdc28 (inactive form), which in turn can lead to G2 delay/stress and therefore many gene expression changes. (PDF) [file pcbi.1007061.s006.pdf]

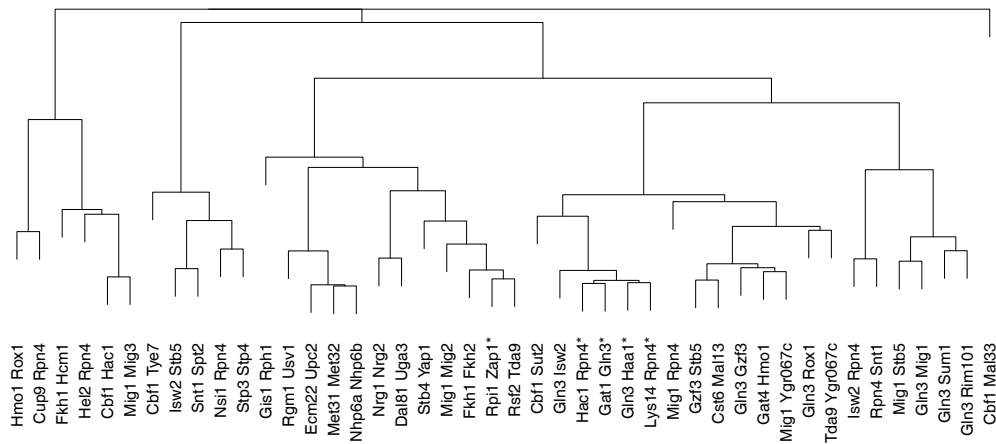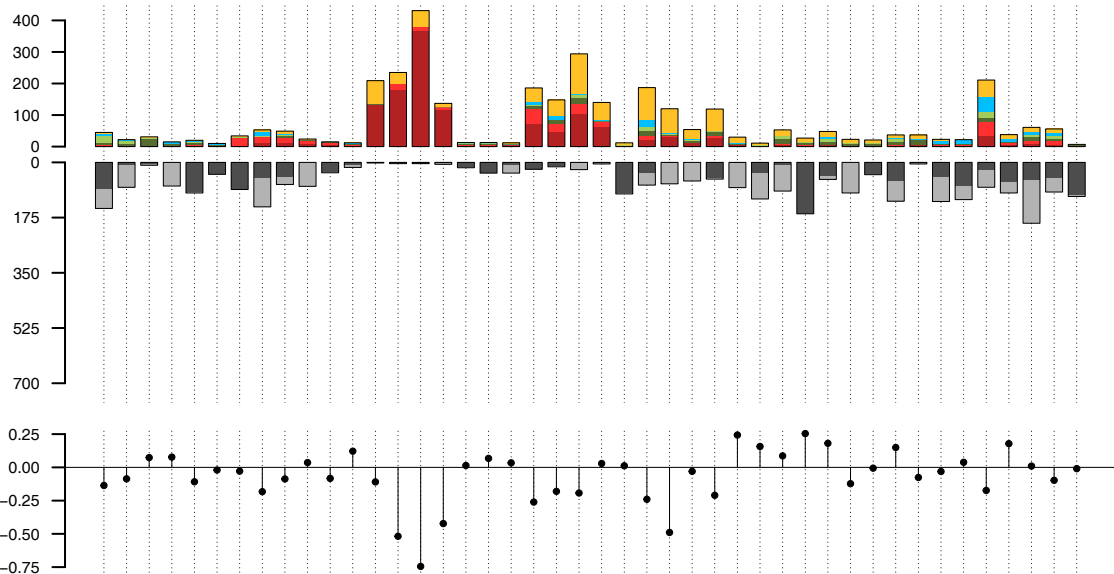

Supplement: S5 Fig — Hierarchical clustering of 44 GSTF pairs according to their genetic interaction effects after slow growth correction. These pairs include both negative and positive genetic interactions. Layout and analysis similar to Fig 2. (PDF) [file pcbi.1007061.s007.pdf]

**A**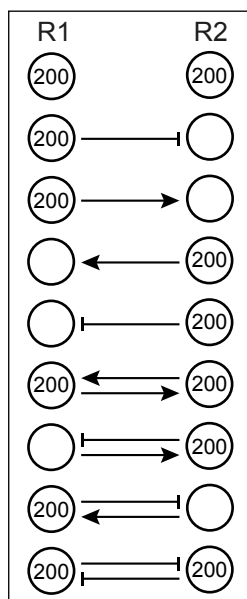**B**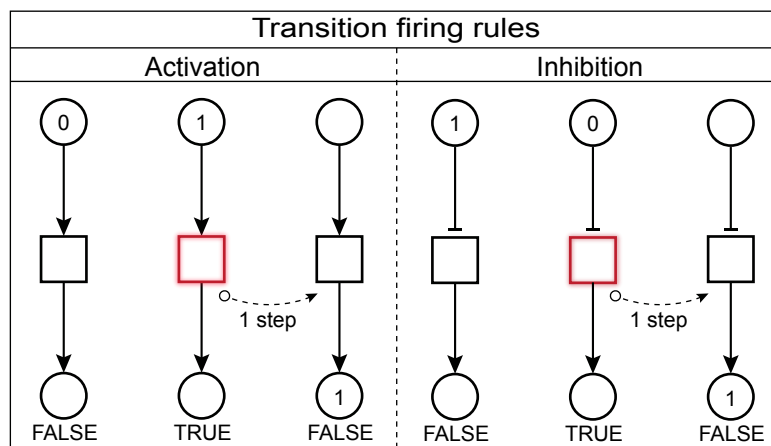

Supplement: S6 Fig — (A) Provided tokens to regulators depending on edges between them. (B) Transition firing rules for activation and inhibition edges depending on the presence of tokens in upstream places. (PDF) [file pcbi.1007061.s008.pdf]
